# Clinical Outcome after the Use of a New Craniocaudal Expandable Implant for Vertebral Compression Fracture Treatment: One Year Results from a Prospective Multicentric Study

**DOI:** 10.1155/2015/927813

**Published:** 2015-01-12

**Authors:** David Noriega, Antonio Krüger, Francisco Ardura, Nils Hansen-Algenstaedt, Frank Hassel, Xavier Barreau, Jörg Beyerlein

**Affiliations:** ^1^Spine-Unit, University Hospital Valladolid, 47008 Valladolid, Spain; ^2^Department of Trauma, Hand and Reconstructive Surgery, Philipps University, 35043 Marburg, Germany; ^3^Department of Spine Surgery, University Medical Center Hamburg-Eppendorf, 20246 Hamburg, Germany; ^4^Department of Spine Surgery, Loretto Clinic, 79100 Freiburg, Germany; ^5^Department of Interventional Neuroradiology, University Hospital Pellegrin, 33000 Bordeaux, France; ^6^Department of Spine Surgery, Bad Bramstedt Clinic, 24576 Bad Bramstedt, Germany

## Abstract

The purpose of this prospective multicentric observational study was to confirm the safety and clinical performance of a craniocaudal expandable implant used in combination with high viscosity PMMA bone cement for the treatment of vertebral compression fractures. Thirty-nine VCFs in 32 patients were treated using the SpineJack minimally invasive surgery protocol. Outcome was determined by using the Visual Analogue Scale for measuring pain, the Oswestry Disability Index for scoring functional capacity, and the self-reporting European Quality of Life scores for the quality of life. Safety was evaluated by reporting all adverse events. The occurrence of cement leakages was assessed by either radiographs or CT scan or both. Statistically significant improvements were found regarding pain, function, and quality of life. The global pain score reduction at 1 year was 80.9% compared to the preoperative situation and the result of the Oswestry Disability Index showed a decrease from 65.0% at baseline to 10.5% at 12 months postoperatively. The cement leakage rate was 30.8%. No device- or surgery-related complications were found. This observational study demonstrates promising and persistent results consisting of immediate and sustained pain relief and durable clinical improvement after the procedure and throughout the 1-year follow-up period.

## 1. Introduction

Vertebral compression fractures (VCFs) are well known to cause pain and disability and influence quality of life [1–3]. One of the most common reasons for a collapse of the vertebra is osteoporosis, thereby often affecting women after menopause, and other patients at risk include those with a history of malignancy or long term use of steroids [4]. Traumatic VCFs are often seen in otherwise healthy subjects due to accidents or fall from heights, causing vertical compression of the spine [5]. Treatment options range from conservative treatment focussing on bed rest and pain medication with or without bracing to invasive treatment aiming at a restoration of the original design of the vertebra [6, 7]. However, by treating patients conservatively pain resolution can be slow and it has been shown that cement augmentation techniques, such as vertebroplasty and kyphoplasty, provide greater and faster pain relief compared with conservative treatment in osteoporotic VCFs [8, 9].

Vertebroplasty, first described by Galibert et al. in 1987 [10], was intended to address the persistent pain, to reduce the fracture by prone positioning of the patient [11], and to stabilize the fractured vertebra in situ by injecting cement through bone biopsy needles. Later on, balloon kyphoplasty was developed to allow surgeons to act directly on the vertebral body. The aim of this procedure was to restore the height of the collapsed vertebral body to address the kyphotic deformity and realign the spine (fracture reduction) and to reduce the risk of cement leakage [12].

These treatments are efficient in reducing pain and improving quality of life. The ligamentotaxis effect can efficiently reduce the cortical ring using the dynamic mobility of the VCF [13]. Kyphoplasty, compared with conservative treatment or vertebroplasty, allows at best an additional restoration of the vertebral height and of the kyphosis angle [14]. Both Verlaan and Voggenreiter have demonstrated that part of the restored height is lost when deflating the balloon prior to the cement injection [11, 15]. Therefore new methods and implants are needed, keeping the height restoration until the stabilizing cement has been injected. Also a direct action on the endplates would be desirable to restore anatomy instead of relying on the ligamentotaxis effect.

A variety of new minimally invasive techniques have been introduced as VCF treatment options over the past years. One of these involves an expandable implant (SpineJack, Vexim Sa, Balma, France) which aims to provide anatomical restoration of the fractured vertebra including cortical ring reduction as well as endplate restoration along with stabilization of the fractured vertebra using a high-viscosity injectable PMMA cement (Cohesion, Vexim Sa, Balma, France). In biomechanical studies comparing SpineJack with balloon kyphoplasty, Krüger et al. could show the superiority of this new technique concerning height restoration and height maintenance [16, 17]. Additionally the surgeon has maximum control of the fracture reduction with the implants only craniocaudal action on the endplates instead of following the path of least resistance.

The primary objective of this first international prospective consecutive multicentre single-arm observational study was to demonstrate the safety and efficacy of this new technique for the treatment of VCF due to osteoporosis and/or trauma.

## 2. Materials and Methods

### 2.1. Patient Population

The data included in this report was collected at seven European clinical sites.

Between November 2009 and March 2010, 32 patients were included and a total of 39 vertebral compression fractures were treated with the new intravertebral implant system.

**Figure 1:**
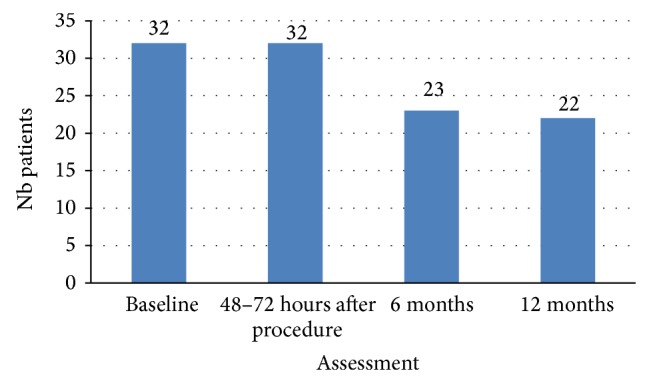
Details of patient recruitment and follow-up.

The patients were given information about the treatment and the follow-up program and signed an informed consent before enrolment. The data was collected at baseline, after 48–72 hours, or at discharge, at 6 and at 12 months. The primary endpoint was to determine the occurrence of cement leakages assessed by X-ray or CT scan. Among the secondary endpoints clinical outcome was measured by reported back pain, quality of life, functional capacity, and the safety of the SpineJack system. Details on patient recruitment and follow-up are given in Figure 1.

Detailed information on gender, age, BMI, and fracture classification has been collected and is presented in Table 1.

Complete data was collected from 21 individuals; 2 patients died and 5 did not show up at any of the follow-ups whereas 4 showed up only once, at 3, 6, or 12 months.

**Figure 2:**
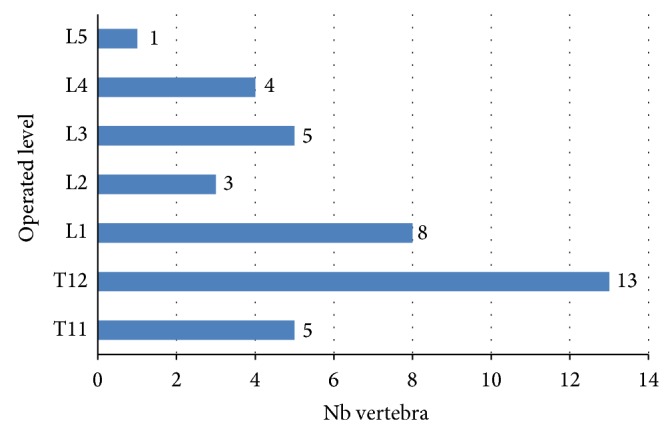
Description of fracture levels.

66.6% of the fractures were located between T11 and L1, 33.4% between L2 and L5. Description of fracture level is provided in Figure 2.

### 2.2. Interventions

All patients underwent a complete physical examination before surgery, including detailed medical history and complete radiographic examination including CT scan to confirm the presence, location, and severity of VCF.

A percutaneous transpedicular approach was used for 97.4% of the patients; in 2,6% an open surgery was performed; this patient was treated with a posterior fixation in combination with the SpineJack procedure. The mean duration of the procedure was 38.3 minutes.

### 2.3. Method

The SpineJack is an intravertebral body implant intended to reduce vertebral compression fractures. The concept is based on the “in situ fracture reduction” principle where the SpineJack implant is expanded in situ to restore the height and anatomy of the vertebral body mechanically.

After restoration high-viscosity polymethylmethacrylate bone cement is injected at low pressure to stabilize the restored vertebra.

The SpineJack is implanted using a percutaneous or minimally invasive posterior surgical approach using surgical tools supplied for this system.

The 5.0 mm implant is made of titanium alloy (Ti6Al4V). The endplate length is 20 mm and the maximum expansion height is 17 mm. The insertion diameter is 5 mm.

**Figure 3:**
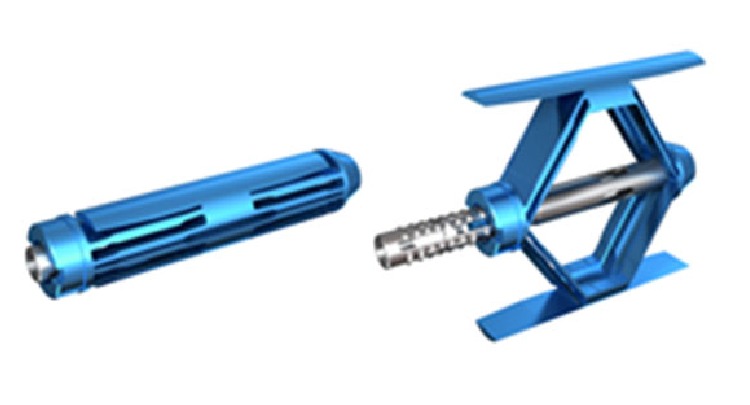
SpineJack unexpanded and after expansion.

SpineJack is inserted into the fractured vertebral body in unexpanded condition, Figure 3, left. After insertion the implant is expanded using a specifically designed tool which is part of the instrumentation kit. It locks into the device and pulls the two ends of the implant towards each other. This longitudinal compression causes the implant to open in craniocaudal direction only due to the machined grooves, Figure 3, right.

**Figure 4:**
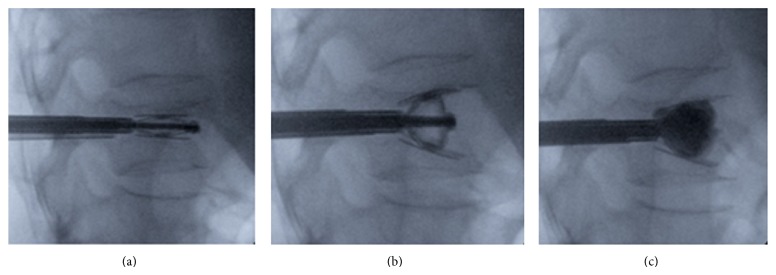
Intraoperative radiographs: (a) expansion of SpineJack implant; (b) fracture reduction; (c) injection of Cohesion bone cement.

Once SpineJack has reached the desired expansion and the reduction has been achieved, the device is left in place inside the restored vertebra and high-viscosity PMMA bone cement is injected into and around the implant, Figure 4. Participating study centres were properly trained in accordance with a preestablished training plan and according to the CE-marked SpineJack Systems Instruction for Use.

### 2.4. Outcome

Outcome was measured using self-completed questionnaires, before surgery, at discharge, and after 6 and 12 months. Pain was assessed by the Visual Analogue Scale (VAS), posture and general self-rated health state by the European Quality of Life score (EQ-5D), and EQ-5D VAS score [18].

Condition specific functional capacity was evaluated by the Oswestry Disability Index (ODI) [19].

Furthermore, the following parameters were recorded by the treating physicians: analgesic intake, duration of hospitalisation, and occurrence of complications. Adverse events (AE) and serious adverse events (SAE) were classified according to the EN ISO 14155 standard during the entire investigation period.

**Figure 5:**
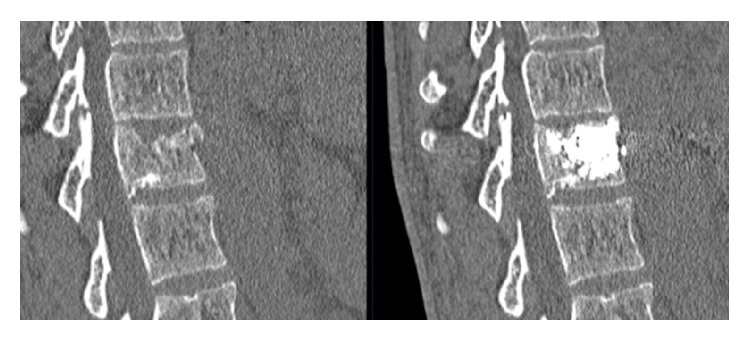
Preop and postop CT scan of the fractured vertebra.

Occurrence of cement extravasation was assessed by either X-ray or CT scan or both, Figure 5.

Fluoroscopic controls monitored the surgical procedure.

## 3. Statistical Methods

Descriptive statistics including quantitative and qualitative parameters were used to describe the number of patients, mean values with standard deviation, median with minimum and maximum values, and percentages of collected data, respectively.

Within group tests were used to see the time effect, that is, evolution between baseline and follow-up visits. Wilcoxon's test or Student's *t*-test for pairwise comparisons was used, depending on the normality of the distribution. *P* value was defined at 0.001.

## 4. Results

The rate of cement leakage observed in this study is 30.8%. The leakages were all asymptomatic and had no consequences on clinical outcome. Five leakages (41.7%) were found in paravertebral veins, 4 (33.3%) in soft tissues, and 3 (25.0%) in the intervertebral disc. Half of the leaks were detected only on CT scan.

**Figure 6:**
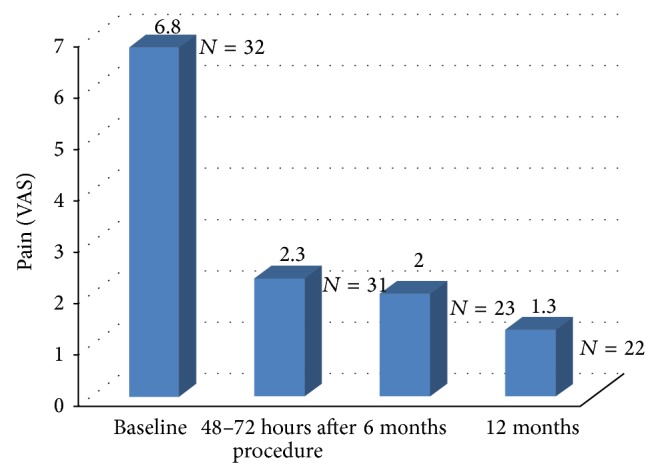
Pain score (VAS) at baseline, after procedure, and at 6-month and at 12-month follow-up.

There was statistically significant, immediate, and long lasting reduction in pain as result of the procedure. The overall improvement in pain (VAS), after treatment (48–72 hours), and at 6 and 12 months was statistically significant (*P* < 0.001). The results are illustrated in Figure 6. A reduction in pain of >1.5 on the VAS scale is considered a meaningful change for back pain, MIC (minimal important change) [20].

The mean improvement between baseline and 48–72 hours after procedure (*n* = 31) is −4.6 (2.6), *P* < 0.001 (Student); the mean improvement between baseline and 12-month follow-up (*n* = 22) is −6.0 (3.4), *P* < 0.001 (Wilcoxon). Thus, the pain reduction is more than 3 times the MIC [20].

The global pain score reduction at 1 year is 80,9% compared to the preoperative situation.

**Figure 7:**
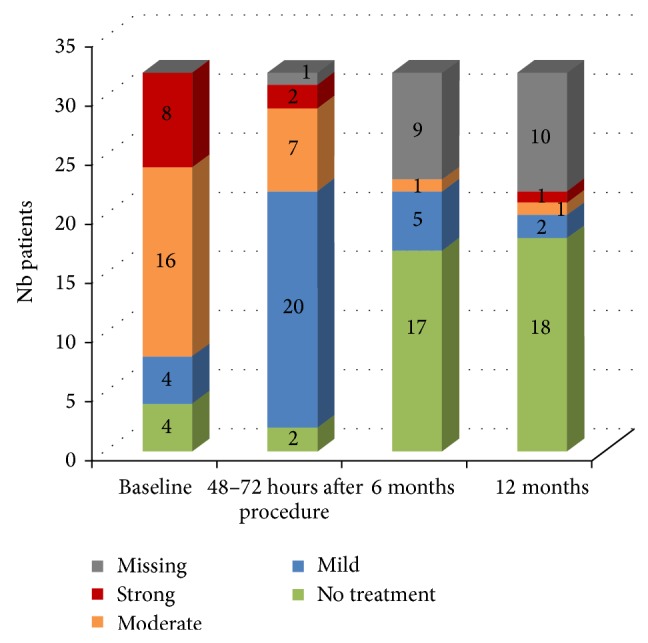
Change in analgesics intake. Mild analgesic medication (including aspirin, nonsteroid anti-inflammatory drugs, paracetamol, and derivatives), moderate analgesic medication (including nonsteroid anti-inflammatory drugs with codeine, propoxyphene with or without codeine, and minor morphine-based analgesics taken once or twice daily), and strong analgesic medication (minor morphine-based analgesics taken three times daily and above, major morphine-based analgesics). 32 patients with baseline data and 22 patients with 12-month data.

A significant reduction in analgesics intake was also observed, Figure 7. At inclusion 8 patients required strong analgesics, postoperatively only 2 patients and only 1 patient at 12 months. The number of patients requiring moderate to strong analgesics decreased from 75.0% at baseline to 9,0% at 12 months.

**Figure 8:**
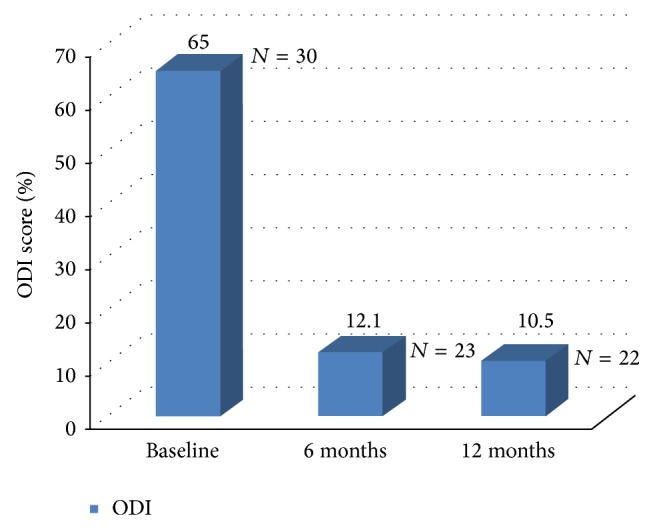
Oswestry Disability Index.

The overall improvement in functional capacity (ODI) was statistically significant, *P* < 0.001 at 6 and at 12 months. The results of the Oswestry Disability Index show a decrease from 65.0% (at baseline) to 10.5% at 12 months postoperatively, which reflects an overall improvement of 83.8% (Figure 8).

**Figure 9:**
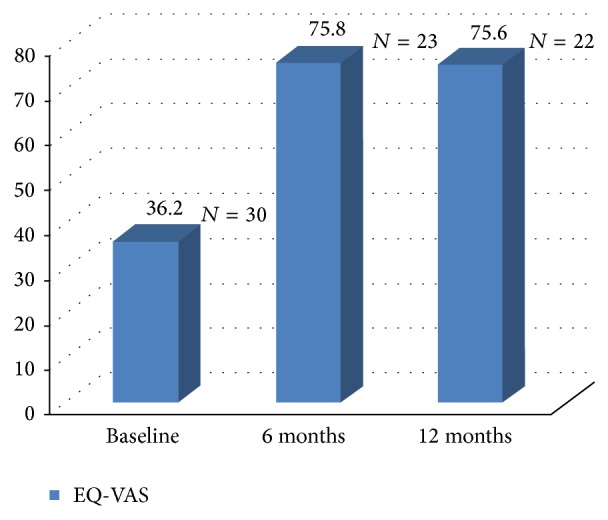
EQ-VAS measures the self-rated health state; a high score signifies “the best health you can imagine”; a low score signifies “the worst health you can imagine.”

We obtained completed quality of life questionnaires for 30 patients at baseline and 22 patients at 12 months. The results of the EQ-VAS show a statistically significant increase of the quality of life, *P* < 0.001, from 36.2% (at baseline) to 75.6% at 12 months postoperatively, Figure 9. The overall improvement was a 52.1% increase of the EQ-VAS at 1 year.

The mean duration of hospitalisation was 3.7 (SD 2.9) days postoperatively with a minimum stay of 2 days and a maximum stay of 17 days. The long hospital stay for one of the patients was due to a SAE (fall in blood pressure/vagal reactions).

Two adverse events (AE) (6.25%) were reported, but neither was implant-related. One patient experienced a fracture of the operated vertebral body at 6-month follow-up, and one experienced a collapse of the disc above as a consequence of the trauma.

There were 7 serious adverse events (SAE) (21.9%) (one patient died due to heart failure 4 months after surgery, and one patient died because of metastatic pancreatic cancer 8 days after procedure, medium cerebral artery infarction, pituitary adenoma, paralysis of diaphragm, fall in blood pressure/vagal reactions, and hospitalisation for degenerative lumbar syndrome with stenosis L3–L5).

None of these were implant- or surgery-related. During this study no device-related complications were reported, and no device had to be removed. There was no surgery-related complication reported during the intervention.

## 5. Discussion

This first clinical investigation with one-year follow-up of a new method to treat vertebral compression fractures indicates promising results.

High-viscosity PMMA cement was used in combination with the implant in this study. While cement leakage occurred in almost a third of the cases, this leakage rate is still low compared to leakage rates shown in numerous publications on vertebroplasty [21–23] and similar to results reported for kyphoplasty, such as those presented by Wardlaw et al. in the FREE study [24].

However, it should be noted that in this study half of the leaks have been identified after studying CT scans, which is a more sensitive method than assessment by radiographs. Yeom et al. [25] did show that more leaks were identified on CT scans than on radiographs by a factor of 1.5. In a recent publication by Dohm et al. [21] leakages were identified on CT scans and the leakage rates presented for balloon kyphoplasty and vertebroplasty were 73% and 82%, respectively.

In this study 22,9% of the fractures were classified as A.3.1 fractures according to the AO Magerl classification. Thaler et al. [26] recently presented the leakage rate for another new augmentation technique for VCF treatment (VBS, Synthes, Oberdorf, Switzerland). However in this study only A.1 fractures were evaluated. In potential instable fractures the cement has a stabilizing function and more cement is used which may contribute to higher leakage rates. Krüger et al. reported a leakage rate of 47.7% in their study on A3.1 burst fractures [27].

Giannitsios et al. [28] showed a strong correlation between cement viscosity and leakage rate in their study model. The authors clearly identified bone cement viscosity as a key parameter influencing leakage, and a critical bone cement viscosity of 350 Pa·s resulted in no leakage.

The viscosity of bone cement used in the cement augmentation procedure is hypothesized to influence the outcome of the procedure in various ways [29, 30].

The reduction of the VAS pain score, 80,9% at the 1-year follow-up, compares favourably with the findings from a recently published study, comparing results from a new vertebral body augmentation technique compared to balloon kyphoplasty [31], presenting pain score reduction of 67% and 68%, respectively, at 1 year. The immediate improvement in pain, documented by the reduction of the VAS pain score, can be explained by the stabilization of the fracture, as seen with fractures in general [32].

The cement will interdigitate with the trabecular bone, and thereby it has a potential to stabilize micromovements in the fractured vertebra and relief pain as well as preserve its mechanical strength. Gennari et al. suggest in a publication that not only the stabilization but also the fracture reduction can be of importance for the pain relief [33].

In this study the response to the reduction/stabilization treatment was immediate and persistent during the 1-year follow-up period. All scores in the EQ-5D (including five subscores) [18] improved significantly except for anxiety/depression. The patients' self-assessed improvement in function and quality of life may be the most important evidence of efficacy. The Oswestry Disability Index has been used for over 25 years and is considered the golden standard [19].

Even more importantly the majority of patients could go back to their normal life demonstrated by significant changes in both EQ-5D and ODI.

An important limitation of the study is the observational study design, including lack of a control group, and the limited follow-up period of 1 year.

Vertebral compression fractures cause pain and disability and influence quality of life. Therefore, it is our opinion that VCFs should be treated surgically [34–36]. Several investigations have demonstrated significant improvement in acute pain and pain-related disability for vertebroplasty and balloon kyphoplasty [9]. The results from this observational study indicate the feasibility by using this new intravertebral implant system.

## 6. Conclusions

This clinical investigation has shown promising results regarding safety and efficacy of the SpineJack procedure when used in combination with high-viscosity PMMA bone cement, with statistically significant improvement in all clinical outcomes including pain, functional capacity, patient's quality of life, and decrease in analgesic intake without causing any serious complications. More importantly the majority of patients could go back to their normal life as shown by significant changes in both EQ-5D and ODI. The cement leakage rate is similar to results reported in the literature for kyphoplasty.

Randomized clinical trials with suitable controls are needed to confirm these first results.

## Figures and Tables

**Table 1:** Patient characteristics.

Characteristics	*n* = 32
	Value
Age (mean ± SD)	71.3 ± 10.7
Female/male *n* (%)	30 (93.7)/2 (6.3)
BMI^*^ (mean ± SD (kg/m^2^))	24.2 ± 3.0
Diagnosis	
Osteoporosis *n*	25
Trauma *n*	7
Number of treated levels	
1 *n* (%)	26 (81.3)
2 *n* (%)	5 (15.6)
3 *n* (%)	1 (3.1)
Fracture age, mean (min/max)	42.2 days (2/244)
Magerl classification^*^	
A1 *n* (%)	16 (45.7)
A2 *n* (%)	11 (31.4)
A3.1 *n* (%)	8 (22.9)
Genant classification	
Wedge fracture *n* (%)	23 (59.0)
Biconcave fracture *n* (%)	12 (30.8)
Crush fracture *n* (%)	4 (10.3)

^*^From 4 cases information is not available.
